# Acceptability of Research and Health Care Visits During the COVID-19 Pandemic: Cross-sectional Survey Study

**DOI:** 10.2196/27185

**Published:** 2021-06-02

**Authors:** Kathryn M Ross, Young-Rock Hong, Rebecca A Krukowski, Darci R Miller, Dominick J Lemas, Michelle I Cardel

**Affiliations:** 1 Department of Clinical and Health Psychology College of Public Health and Health Professions University of Florida Gainesville, FL United States; 2 Center for Integrative Cardiovascular and Metabolic Diseases University of Florida Gainesville, FL United States; 3 Department of Health Outcomes and Biomedical Informatics College of Medicine University of Florida Gainesville, FL United States; 4 Department of Preventive Medicine College of Medicine University of Tennessee Health Science Center Memphis, TN United States

**Keywords:** COVID-19, health care access, telehealth, research recruitment, telemedicine, belief, access, willingness, cross-sectional, survey

## Abstract

**Background:**

The COVID-19 pandemic has had a widespread impact on attendance in biomedical research and health care visits.

**Objective:**

This study aimed to identify when and how American adults might feel comfortable about resuming in-person research and health care visits.

**Methods:**

Cross-sectional questionnaire data were collected from 135 adults (age: median 48 years; women: n=113, 83.7%; White participants: n=92, 68.2%) who were engaged in health-related research.

**Results:**

More than half of the respondents (65/122, 53.3%) felt that the COVID-19 pandemic positively affected their desire to participate in research. Although 73.6% (95/129) of respondents also indicated a willingness to attend in-person health care visits while Centers for Disease Control and Prevention (CDC) guidelines are implemented, 85.8% (109/127) indicated a willingness to attend in-person, outdoor visits, and 92.2% (118/128) reported a willingness to attend drive-through visits (with CDC guidelines implemented during both visit types). Videoconferencing was the most preferred format for intervention visits; however, adults over the age of 65 years preferred this format less than younger adults (*P*=.001).

**Conclusions:**

Researchers and clinicians should continue to provide opportunities for continuing the conduction of remote-based interventions while enforcing CDC guidelines during in-person visits.

## Introduction

Lockdown and stay-at-home orders that were enacted to contain the spread of COVID-19 [1] have disrupted biomedical research and health care [2,3]. There has been a rapid increase in the adoption of telehealth methods that provide remote care delivery [4,5], which has offset some of the impacts of the COVID-19 pandemic [6]; however, it remains important to understand how to resume the provision of research and clinical care in a manner that individuals feel is safe. Identifying these factors could provide insights into feasible and acceptable approaches to conducting research and health care visits both during the ongoing pandemic surge and, importantly, during future postpandemic recovery [7]. Thus, this study investigated the acceptability of in-person and remote research and health care visits during the COVID-19 pandemic in order to characterize when and how American adults would feel comfortable about resuming research and health care–related clinic visits.

## Methods

This study was a cross-sectional analysis of US adults who were enrolled in biomedical research studies. An email describing this study and a link for participating in the web-based survey was sent to 250 adults who had previously completed a survey that assessed COVID-19 impacts on research participation and mental health outcomes [8], and additional participants were recruited through professional networks. Networks included colleagues of study investigators who were conducting behavioral intervention trials across the United States. They were informed via email and social media (Facebook and Twitter) to share the study information and survey link with participants who were enrolled in their studies. Potential respondents who clicked the survey link were provided with a description of this study and were asked to provide informed consent before completing the survey. Consent was obtained and data were collected via REDCap (Research Electronic Data Capture; Vanderbilt University) [9] between September 17 and October 17, 2020.

The 87-item questionnaire, which was developed by the study team, was used to collect sociodemographic information (ie, age, gender, race and ethnicity, educational attainment, and household income), query respondents about the number of underlying health conditions that were suspected to increase COVID-19 severity [10], and ask other questions about physical and mental health and research participation. This study used data collected from 10 items that focused on engagement with research and the willingness to attend research and health care visits (Multimedia Appendix 1). The University of Florida Institutional Review Board provided ethical approval for this study.

Descriptive statistics were computed to describe research participation and health care engagement. In total, 3 items for querying respondents about their beliefs regarding participating in research and whether the COVID-19 pandemic has positively or negatively affected their desire to participate in research were scored on 5-point, Likert-style scales (Multimedia Appendix 1). For analysis purposes, responses to each item were converted into binary indicators; “Not at all” was categorized as “No,” and the responses “A little bit,” “Moderately,” “Quite a bit,” and “Extremely” were categorized as “Yes.” Furthermore, 4 items for querying respondents about their willingness to attend various types of health care visits were scored on a 3-point scale. Similarly, binary variables were used to indicate whether a respondent would feel comfortable with each health care visit type (ie, in-person visits, outdoor visits, and drive-through clinic visits). Chi-square and Fisher exact tests were used for bivariate analyses involving participants’ age, gender, and race. Analyses were conducted by using SAS (Statistical Analysis System) version 9.4 (SAS Institute), and statistical significance was assessed based on a 2-sided *P* value of <.05. Study findings were reported by using the STROBE (Strengthening the Reporting of Observational Studies in Epidemiology) guidelines.

## Results

Responses were collected from 135 adults participating in biomedical research. Table 1 provides the full sample’s characteristics (see Multimedia Appendix 2 for a correlation table between these variables). The median age of respondents was 48 years, and the sample was predominately female (113/135, 83.7%) and Non-Hispanic White (92/135, 68.2%). Moreover, the sample was highly educated (at least a college degree: 103/135, 76.3%), had generally high incomes (respondents reporting a household income of ≥US $75,000 per year: 77/135, 57.1%), and was well-insured (respondents reporting having private or public health insurance: 127/135, 94.1%).

**Table 1:** Sample characteristics.

Characteristic		Value
Age (years), median (IQR)		48 (38-57)
**Age group (years), n (%)**		
	18-39	40 (29.6)
	40-54	50 (37)
	55-64	28 (20.7)
	≥65	17 (12.6)
**Gender, n (%)**		
	Women	113 (83.7)
	Men	22 (16.3)
**Race and ethnicity, n (%)**		
	Non-Hispanic White	92 (68.2)
	Non-Hispanic Black	11 (8.1)
	Hispanic	7 (5.2)
	Asian	2 (1.5)
	Other or multiple races	23 (17)
**Education, n (%)**		
	High school or less	6 (4.4)
	Some college	26 (19.3)
	College graduate	31 (23)
	Some graduate or professional	13 (9.6)
	Graduate or professional degree	59 (43.7)
**Household income (US $), n (%)**		
	<25,000	19 (14.1)
	25,000-49,999	23 (17)
	50,000-74,999	16 (11.9)
	75,000-99,999	21 (15.6)
	≥100,000	56 (41.5)
**Health insurance, n (%)**		
	Private	92 (68.2)
	Public	35 (25.9)
	Uninsured	8 (5.9)
**Number of underlying conditions, n (%)**		
	0	98 (72.6)
	1	29 (21.5)
	≥2	8 (5.9)

Table 2 presents participants’ beliefs and willingness to attend research and health care–related clinic visits. Respondents were more likely to indicate that the COVID-19 pandemic positively (vs negatively) impacted their desire to participate in research (65/122, 53.3% vs 49/122, 40.2%; *P*=.04). Moreover, a majority of respondents (77/120, 64.2%) indicated that they did not believe that such participation put them at greater risk of contracting COVID-19. The most preferred methods of engaging in behavioral interventions included videoconferencing (60.7%) and in-person sessions (53.3%). Regarding future research participation, respondents were most interested in engaging in clinical (108/135, 80%) and public health research (85/135, 63%) and were least interested in vaccine development research (59/135, 43.7%).

**Table 2:** Research participants’ preferences for engaging in research and clinical care during the COVID-19 pandemic.

Questions and responses				Value, n (%)^a^
**Research participation**				
	**Do you believe that participating in research puts you more at risk for COVID-19?**			
		No (not at all)	77 (64.2)	
		Yes (a little bit to extremely)	43 (35.8)	
	**Has COVID-19 positively affected your desire to participate in research?**			
		No (not at all)	57 (46.7)	
		Yes (a little bit to extremely)	65 (53.3)	
	**Has COVID-19 negatively affected your desire to participate in research?**			
		No (not at all)	73 (59.8)	
		Yes (a little bit to extremely)	49 (40.2)	
	**What type of research would you be interested in participating in at a future time? (multiple responses allowed)**			
		Public health (eg, hand washing to prevent flu)	85 (63)	
		Emergency preparedness (eg, preparing for a natural disaster)	68 (50.4)	
		Vaccine development (eg, COVID-19 vaccine development)	59 (43.7)	
		Clinical research (eg, studies that help you improve your own health)	108 (80)	
**Clinic visit**				
	**Attending in-person clinic visits during the COVID-19 pandemic while adhering to social distancing, sanitation, and mask-wearing protocols**			
		Not comfortable	34 (26.4)	
		Somewhat or very comfortable	95 (73.6)	
	**Attending in-person, outdoor clinic visits during the COVID-19 pandemic while adhering to social distancing, sanitation, and mask-wearing protocols**			
		Not comfortable	18 (14.2)	
		Somewhat or very comfortable	109 (85.8)	
	**Attending drive-through clinic visits during the COVID-19 pandemic with masks**			
		Not comfortable	10 (7.8)	
		Somewhat or very comfortable	118 (92.2)	
	**Attending drive-through clinic visits during the COVID-19 pandemic without masks**			
		Not comfortable	76 (59.8)	
		Somewhat or very comfortable	51 (40.2)	
	**At what point would you be willing to go back to in-person clinic visits? (multiple responses allowed)**			
		When there is a COVID-19 vaccine	41 (30.4)	
		When there is a medication for effectively treating COVID-19	30 (22.2)	
		When cases have decreased in my area for 2 weeks or more	23 (17)	
		When hospitals have the capacity to treat cases	10 (7.4)	
		I already feel comfortable attending an in-person clinic visit	71 (52.6)	
		I don’t think I will feel comfortable going to an in-person visit until there are no cases of COVID-19 in the United States	8 (5.9)	
	**What is your preferred way of engaging in treatment if enrolled in behavioral intervention? (multiple responses allowed)**			
		In person	72 (53.3)	
		Phone	49 (36.3)	
		Videoconferencing platforms (eg, Zoom and Google Hangouts)	82 (60.7)	
		Other platforms (eg, Slack, WeChat, and GroupMe)	33 (24.4)	

^a^Sample size is not equal across questions due to missing responses.

Almost three-quarters of respondents (95/129, 73.6%) felt comfortable with attending in-person, indoor clinic visits, and over 85% felt comfortable with attending outdoor (109/127, 85.8%) and drive-through (118/128, 92.2%) clinic visits while adhering to Centers for Disease Control and Prevention (CDC) guidelines (social distancing, sanitation, and mask wearing; Table 2). Although respondents indicated the highest level of comfort with attending drive-through visits while wearing a mask (118/128, 92.2%), fewer respondents were comfortable with attending drive-through visits without masks (51/127, 40.2%). Over half of the respondents (71/135, 52.6%) reported a willingness to attend in-person clinic visits at the time the survey was conducted; one-third (41/135, 30.4%) reported a willingness to attend in-person clinic visits when there is a COVID-19 vaccine, and over 20% (30/135) reported a willingness to attend in-person clinic visits when there is a medication for effectively treating COVID-19. Fewer participants reported considering local or national case counts (23/135, 17% and 8/135, 5.9%, respectively) or hospital capacity for treating COVID-19 cases (10/135, 7.4%) when determining their willingness to attend in-person clinic visits.

There were no significant differences between men and women or between White participants and people of color in terms of responses for any survey items (all *P* values were >.05); however, younger adults (aged 18-64 years) were significantly more likely to report feeling comfortable with videoconferencing platforms than adults over the age of 65 (78/118, 66.1% vs 4/17, 23.5%; *P*=.001).

## Discussion

Research participants felt that engaging in biomedical research studies did not increase their personal risk of contracting COVID-19 and were interested in future research activities. Interestingly, over half of respondents indicated that the COVID-19 pandemic positively impacted their desire to participate in research (65/122, 53.3%), and fewer indicated that the COVID-19 pandemic had a negative impact (49/122, 40.2%). Although we do not have additional data to explain this finding, it is possible that the greater news coverage of biomedical research during the pandemic has increased individuals’ appreciation for this scientific process [11] and increased individuals’ interest in participating in research. It also may be possible that some individuals (especially younger adults and those with high incomes [12,13]) have experienced increased scheduling flexibility due to the pandemic (eg, due to cancelled travel and events and restrictions on many leisure-time activities [14]) and thus may have more free time to participate in research.

With the ongoing pandemic, most respondents reported a willingness to attend in-person, health care–related clinic visits while adhering to CDC guidelines (95/129, 73.6%); however, even more were comfortable with outdoor (109/127, 85.8%) and drive-through (118/128, 92.2%) formats. Our results also suggested that the availability of vaccines and effective COVID-19 treatments may improve individuals’ willingness to resume attending in-person clinic visits. Overall, videoconferencing was the most preferred format. Videoconferencing offers greater flexibility in scheduling compared to in-person visits, and this format can also retain the visual cues (eg, eye contact and body language) that are lost in phone-based delivery formats [15]. The increased use of smartphones and other mobile devices [16] coupled with advances in mobile internet speeds have made videoconferencing an accessible delivery format for a large proportion of the population, although access to unlimited data packages and poor coverage (eg, in rural areas) still remain substantial barriers for many people [17]. Considerations should also be made regarding the technology literacy of target populations (eg, our results demonstrated that adults over the age of 65 preferred this format less than younger adults).

Taken together with other findings that support the role of telehealth approaches in health care delivery [15], our results suggest that researchers and clinicians should provide opportunities for continuing the conduction of remote-based intervention after the pandemic. As research and health care centers move beyond stopgap telehealth approaches, such opportunities will require the development of sustainable, secure telehealth systems that can link to existing medical record networks, provide access to affiliate providers, and provide critical data security and patient privacy [7].

The limitations to this study included the use of a convenience sample of biomedical research participants that consisted predominately of highly educated White women with health insurance. Although this sample is generally reflective of research populations within the nutrition and obesity intervention fields [18,19], this limited our results’ generalizability to other populations. The fact that respondents were already participating in biomedical research may have also resulted in bias, as personal experiences with web-based or in-person research may affect the perceived acceptability of these formats and promote a greater willingness to participate in future research studies. Further, due to the descriptive nature of the analysis, additional studies (including those with larger and more generalizable samples) are necessary to replicate our results and to explore other facilitators and barriers to attending health-related research and clinic visits. Finally, there were no approved vaccines for COVID-19 at the time when the questionnaire was distributed; however, several vaccines have since received approval in the United States [20,21], with distribution starting in early December 2020 [22]. Thus, future studies should assess whether the actual (vs hypothetical) availability of these vaccines affects individuals’ willingness to attend research and clinic appointments (especially given the unexpectedly high prevalence of vaccine hesitancy during the initial vaccine rollouts [23]). Despite these limitations, the results from this study provide timely evidence for informing practitioners and researchers about how to reopen and resume research and clinic operations.

## Appendix

Multimedia Appendix 1 Survey questions used in this study.

Multimedia Appendix 2 Correlation matrix between the sample's characteristics.

## Abbreviations

CDC: Centers for Disease Control and Prevention
NHLBI: National Heart, Lung, and Blood Institute
NIDDK: National Institute of Diabetes and Digestive and Kidney Diseases
REDCap: Research Electronic Data Capture
SAS: Statistical Analysis System
STROBE: Strengthening the Reporting of Observational Studies in Epidemiology
