# Supplementary material for: Acceptability of Research and Health Care Visits During the COVID-19 Pandemic: Cross-sectional Survey Study
Source: JMIR Form Res. 2021 Jun 2;5(6):e27185. doi: 10.2196/27185 (PMC8174557; doi:10.2196/27185)
Supplement: Multimedia Appendix 1 [file formative_v5i6e27185_app1.docx]

**Multimedia Appendix 1.** Survey questions used in this study.

**COVID-19 Experience**

***We are interested in understanding how coronavirus (COVID-19) has impacted your lifestyle and your behavioral health research participation.***

**1. What is your age (in years)? ____**

**2. What is your gender?**

1. Woman
2. Man
3. Non-binary
4. Other
5. Prefer not to answer

**3. What is your highest level of education completed?**

1. 8^th^ grade or less
2. Some high school
3. High school diploma/GED
4. Some college
5. Associate degree
6. Completed tech or vocational school
7. College graduate
8. Some graduate or professional school
9. Graduate or professional degree
10. Prefer not to answer

**4. What is the income category that best represents your total household income for the previous year?**

1. Less than $5,000
2. $5,000-$9,999
3. $10,000-$14,999
4. $15,000-$19,999
5. $20,000-$24,999
6. $25,000-$29,999
7. $30,000-$34,999
8. $35,000-$39,999
9. $40,000-$44,999
10. $45,000-$49,999
11. $50,000-$54,999
12. $55,000-$59,999
13. $60,000-$74,999
14. $75,000-$99,999
15. $100,000-$124,999
16. $125,000-$149,999
17. $150,000-$199,999
18. $200,000 or more
19. I don’t know
20. Prefer not to answer

**5. What is your ethnicity?**

1. Hispanic/Latino
2. Not Hispanic/Latino
3. Prefer not to answer

**6. What is your race?**

- 1. White
  2. Black or African American
  3. Asian
  4. American Indian or Alaskan Native
  5. Middle Eastern
  6. Native Hawaiian or Pacific Islander
  7. Multiple Races
  8. Other race (please specify):
  9. Prefer not to answer

**7. Do you currently have any of the following? *(Check all that apply.)***

- 1. Respiratory issues
  2. Diabetes
  3. Hypertension
  4. Cancer
  5. I have not been diagnosed with any of the above
  6. Prefer not to answer

**8. What type of health insurance do you have? *(Check all that apply.)***

- 1. Private health insurance
  2. Medicare
  3. Medigap
  4. Medicaid
  5. Children’s Health Insurance Program
  6. Military related health care: TRICARE (CHAMPUS) /VA Healthcare/ CHAMP-VA
  7. Indian Health Service
  8. State-sponsored health plan
  9. Other government program
  10. No coverage of any type
  11. I don’t know
  12. Prefer not to answer

**Research Intervention Participation**

**9. Do you believe that participating in research puts you more at risk for COVID-19?**

a. Not at all

b. A little bit

c. Moderately

d. Quite a bit

e. Extremely

**10. Has COVID-19 positively affected your desire to participate in research?**

a. Not at all

b. A little bit

c. Moderately

d. Quite a bit

e. Extremely

**11. Has COVID-19 negatively affected your desire to participate in research?**

a. Not at all

b. A little bit

c. Moderately

d. Quite a bit

e. Extremely

**12. What type of research would you be interested in participating in at a future time? *(Check all that apply.)***

a. Public health (e.g., hand washing to prevent flu)

b. Emergency preparedness (e.g., preparing for a natural disaster)

c. Vaccine development (e.g., COVID-19 vaccine development)

d. Clinical research (e.g., studies that help you improve your own health)

e. Other Please list: ____________

**How comfortable would you feel doing the following activities?:**

**13. Attending in-person clinic visits during the COVID-19 pandemic while adhering to social distancing, sanitation, and mask-wearing protocols?**

1. Not comfortable
2. Somewhat comfortable
3. Very comfortable

**14. Attending in-person outdoor clinic visits during the COVID-19 pandemic while adhering to social distancing, sanitation, and mask-wearing protocols?**

1. Not comfortable
2. Somewhat comfortable
3. Very comfortable

**15. Attending drive-thru clinic visits during the COVID-19 pandemic with masks?**

1. Not comfortable
2. Somewhat comfortable
3. Very comfortable

**16. Attending drive-thru clinic visits during the COVID-19 pandemic without masks?**

1. Not comfortable
2. Somewhat comfortable
3. Very comfortable

**17. At what point would you be willing to go back to in-person clinic visits? *(Check all that apply.)***

1. When there is a COVID-19 vaccine
2. When there is a medication to effectively treat COVID-19
3. When cases have decreased in my area for two weeks or more
4. When hospitals have the capacity to treat cases
5. I already feel comfortable attending an in-person clinic visit
6. I don’t think I will feel comfortable going to an in-person visit until there are no cases of COVID-19 in the United States

**18. What is your preferred way of engaging in treatment if enrolled in behavioral intervention? *(Check all that apply.)***

1. In-person
2. Phone
3. Zoom
4. Slack
5. Text
6. Microsoft Teams
7. Google Hangouts
8. Private group chats (i.e., WeChat, GroupMe, etc.)
