# Supplementary material for: Acceptability of Research and Health Care Visits During the COVID-19 Pandemic: Cross-sectional Survey Study
Source: JMIR Form Res. 2021 Jun 2;5(6):e27185. doi: 10.2196/27185 (PMC8174557; doi:10.2196/27185)
Supplement: Multimedia Appendix 2 [file formative_v5i6e27185_app2.docx]

**Multimedia Appendix 2.** Correlation matrix between the sample's characteristics.

| **Characteristic** | **M** | **SD** | **1** | **2** | **3** | **4** | **5** | **6** | **7** |
| --- | --- | --- | --- | --- | --- | --- | --- | --- | --- |
| **1. Age** | 47.84 | 13.89 | 1 |  |  |  |  |  |  |
| *P*-value | - | - | - |  |  |  |  |  |  |
| **2. Gender: Male** | 0.16 | 0.37 | 0.234 | 1 |  |  |  |  |  |
| *P*-value | - | - | .006 | - |  |  |  |  |  |
| **3. Race/Ethnicity: Minority** | 0.32 | 0.47 | 0.048 | 0.000 | 1 |  |  |  |  |
| *P*-value | - | - | .58 | .99 | - |  |  |  |  |
| **4. Educational level** | 3.69 | 1.32 | -0.147 | -0.018 | -0.044 | 1 |  |  |  |
| *P*-value | - | - | .09 | .84 | .62 | - |  |  |  |
| **5. Income level** | 3.53 | 1.51 | 0.039 | 0.044 | -0.147 | 0.319 | 1 |  |  |
| *P*-value | - | - | .65 | .62 | .09 | <.001 | - |  |  |
| **6. Uninsured** | 0.06 | 0.24 | -0.04 | -0.026 | -0.104 | -0.107 | -0.131 | 1 |  |
| *P*-value | - | - | .64 | .77 | .23 | .22 | .13 | - |  |
| **7. Number of conditions** | 0.33 | 0.59 | 0.302 | 0.092 | 0.182 | -0.183 | -0.067 | -0.09 | 1 |
| *P*-value | - | - | <.001 | .29 | .04 | .03 | .44 | .30 | - |
